# Upper limb activity of twenty myoelectric prosthesis users and twenty healthy anatomically intact adults

**DOI:** 10.1038/s41597-019-0211-6

**Published:** 2019-10-10

**Authors:** Alix Chadwell, Laurence Kenney, Malcolm Granat, Sibylle Thies, Adam Galpin, John Head

**Affiliations:** University of Salford, Health Sciences Research Centre, Salford, United Kingdom

**Keywords:** Outcomes research, Databases

## Abstract

The upper limb activity of twenty unilateral upper limb myoelectric prosthesis users and twenty anatomically intact adults were recorded over a 7-day period using two wrist worn accelerometers (Actigraph, LLC). This dataset reflects the real-world activities of the participants during their normal day-to-day routines. Participants included students, working adults, and retirees recruited from across the United Kingdom. This dataset offers a potential wealth of knowledge into a poorly understood cohort. The raw unprocessed data files and the activity count data exported from the Actilife software are provided. We also provide a non-wear algorithm developed for the removal of prosthesis non-wear periods and resulting activity count data corresponding to prothesis wear periods. Finally, we have included the transposed activity diaries provided by the participants. Analysis to date has primarily involved assessment of the symmetry of upper limb activity, however, there is potential to undertake additional analysis such as understanding the differences in the way a prosthesis is used compared to an anatomical arm.

## Abstract (summary)

**Table:** 

Measurement(s)	acceleration • sleep duration • Prosthesis wear time • Sensor wear time
Technology Type(s)	Accelerometer • Subject Diary
Sample Characteristic - Organism	Homo sapiens
Sample Characteristic - Location	United Kingdom

Machine-accessible metadata file describing the reported data: 10.6084/m9.figshare.9879596

## Background & Summary

Statistics relating to the prevalence of upper limb absence and provision of prostheses are poor^1^. In the UK, based on informed discussions with upper limb prosthetists/occupational therapists, we estimate that there are approximately 800–1000 people registered to the 43 NHS limb centres who have been prescribed with a myoelectric prosthesis^2^ (a myoelectric prosthesis contains motors which are controlled using the naturally generated electrical signals produced during muscle contractions). It is believed that this population may encompass those who wear the prosthesis all day, every day, through to those who have completely rejected the device. The reasons for wear/non wear and use/non-use decisions by users are difficult to unpick in the absence of high quality, real world usage data. However, until very recently^1^, there has been no objective data on upper limb activity in prosthesis users.

For a person with unilateral upper limb absence, over-reliance on the intact side of the body can lead to overuse injuries^3–5^. It is widely believed that by providing the person with a functional prosthesis, such as a myoelectric prosthesis, offering control over movement of the prosthetic digits, reliance on the anatomically intact side may reduce. Many of the current methods for evaluating the use of upper limb prostheses are time consuming for clinicians, and recent research has shown that the results of upper limb assessments within labs/clinics may not correlate with how a prosthesis is used in the real-world^6^. Activity monitors offer an objective, simple and time-efficient way to evaluate periods of prosthesis wear and use. In this paper we publish a real world upper limb activity dataset on myoelectric prosthesis users (and others), collected as part of a larger project^6^.

The upper limb activity dataset was recorded using commercial, wrist worn, tri-axial accelerometers (Actigraph, LLC). Twenty unilateral trans-radial myoelectric prosthesis users, and twenty anatomically intact adults took part in the study. For each participant the data were recorded from each wrist over a 7-day period, whilst they went about their normal day-to-day activities. For the prosthesis users, one of the two sensors remained on the wrist of their myoelectric prosthesis, regardless of whether they were wearing the prosthetic arm at the time. We define activity as accelerations of the forearms exceeding thresholds sufficient to generate an ‘activity count’^7^ on any of the axes.

We previously published methods developed to quantify the symmetry of upper limb activity over time and to display this data graphically using histograms and spiral plots^8^. Our initial analysis of this upper limb activity dataset using these methods has been published in an associated journal^6^. However, further analysis of these data is possible and may be beneficial. For example, in Chadwell *et al*.^8^ we presented only graphical description of the time-series data and there is potential for further analysis of this aspect of the dataset, through for example, fractal analysis^9^ (as suggested by Raoul Bongers – University of Groningen – 2019 – Personal communication). Further, additional analyses of the dataset, based on, for example different epoch lengths, or different methods for the interpretation of movement intensity^1,10^ may also be warranted. By making the full upper limb activity dataset publicly available through figshare^11^ we present others with opportunities to undertake their own analysis. In this data-descriptor we provide the information required for additional analysis. Specifically, we provide the raw unprocessed data (30 Hz) from the accelerometers recorded over 7 days. Note that this raw data includes periods when the monitors were not worn by the participants. Self-reported wear times are included to aid interpretation of the data. We also provide downsampled, epoched, filtered data exported from the Actilife software in the form of activity counts. We have included files with 1 s and 60 s epochs, downloaded with and without the additional Low Frequency Extension filter^12^. Finally, we have included data (based on the 60 s epoch data with the LFE filter applied) which has been passed through our prosthesis non-wear algorithm.

In the supplementary material to our earlier Scientific Reports paper^6^, we described the development of this non-wear algorithm, which removes prosthesis non-wear periods based on the accelerometer signals. In this data descriptor we provide the necessary detail to allow both for the replication of our analysis and the development of a more advanced non-wear algorithm. To this end we have we have provided the Matlab code for this non-wear algorithm, as well as the post-processed data detailing the calculated prosthesis wear status (worn/not-worn) for each epoch. We have also included in the dataset the self-reported prosthesis removal, sensor removal, and sleep times provided by the prosthesis users. Please note that at present we have only undertaken detailed testing^6^ of our algorithm using 60 s epoch data, but all of the information required to run analyses using different epoch lengths are provided in the upper limb activity dataset^11^.

## Methods

These methods are expanded versions of descriptions in our related work^6^, where we have published the results of our initial analysis of this dataset; note that the participant demographic summary is the same for both manuscripts.

### Participants

Twenty participants (14 male, 6 female) with unilateral upper limb absence at a trans-radial level were recruited from six (4 NHS, 2 University) sites across the UK. All participants had a single degree of freedom myoelectric prosthesis (e.g. Steeper Select or Ottobock DMC Plus/VariPlus/Sensor Speed). Full metadata is provided with the dataset^11^.

The age of the prosthesis users ranged from 18 to 75 years (median age 54 years). Eleven people had congenital limb absence (6 Right/5 Left), and nine had an amputation (6 Right/3 Left); six of the amputations had occurred on the dominant side. Time since amputation ranged from 8–47 years (median 24 years). Time since prescription of a myoelectric prosthesis ranged from 1.5–39 years (median 20 years).

A group of twenty anatomically intact participants (9 male, 11 female, age 23–61, median age 41, 3 left handed) with no upper limb impairments were also recruited through the University of Salford.

Ethical approval for this study was granted by the University of Salford School of Health Sciences Research Ethics committee (REF: HSCR 16–25), by the University of Strathclyde Department of Biomedical Engineering Ethics Committee (DEC.BioMed.2017.220) and through the NHS IRAS system (IRAS Project ID: 193794). Informed consent was gained from all participants.

### Sensors

Each participant was provided with two identical Actigraph activity monitoring sensors from the GT3X range (GT3X + , wGT3X, wGT3X-BT), one labelled to be worn on the right wrist and the other labelled for the left wrist. The specific sensors for each data record are detailed in the metadata and in the file titles. These sensors record accelerations along three axes with a dynamic range of +/−6g (+/−8g for the wGT3X-BT) and a sensitivity of 2.93 mg (3.91 mg for the wGT3X-BT). Acceleration is sampled by a 12-bit analogue to digital convertor at user selectable rates between 30 Hz and 100 Hz (for this study they were all set at 30 Hz). The data is stored directly onto non-volatile flash memory in a raw, non-filtered/accumulated format in units of gravity (g).

### Activity diary

Each participant was asked to complete an activity diary to assist with the interpretation of the accelerometer data. Information collected were the times when the participant went to sleep/woke up, when they removed the prosthesis/put the prosthesis on, and when they removed the sensors/put the sensors on.

### Sensor initialisation

**Fig. 1:**
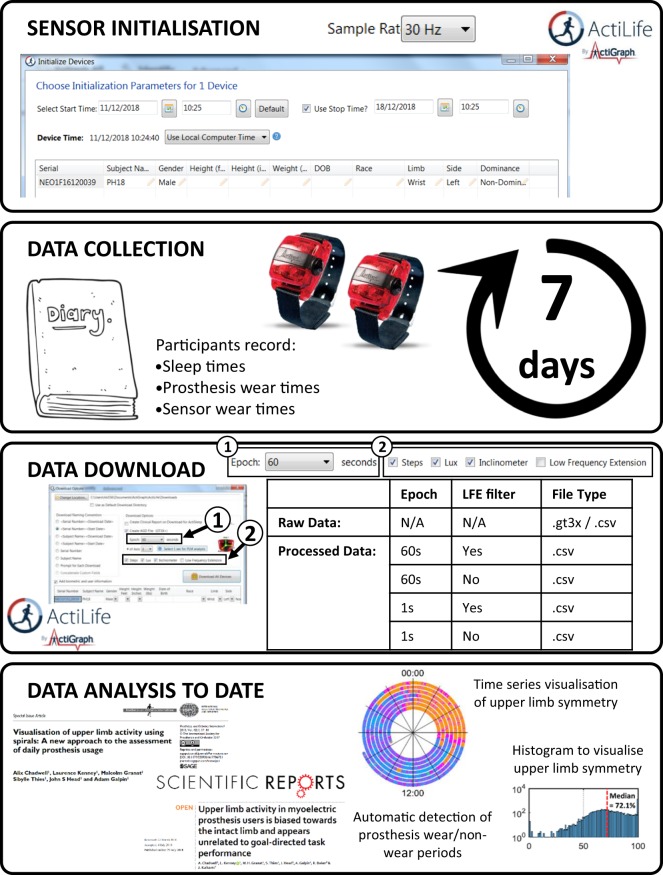
Schematic for data collection and processing.

**Fig. 2:**
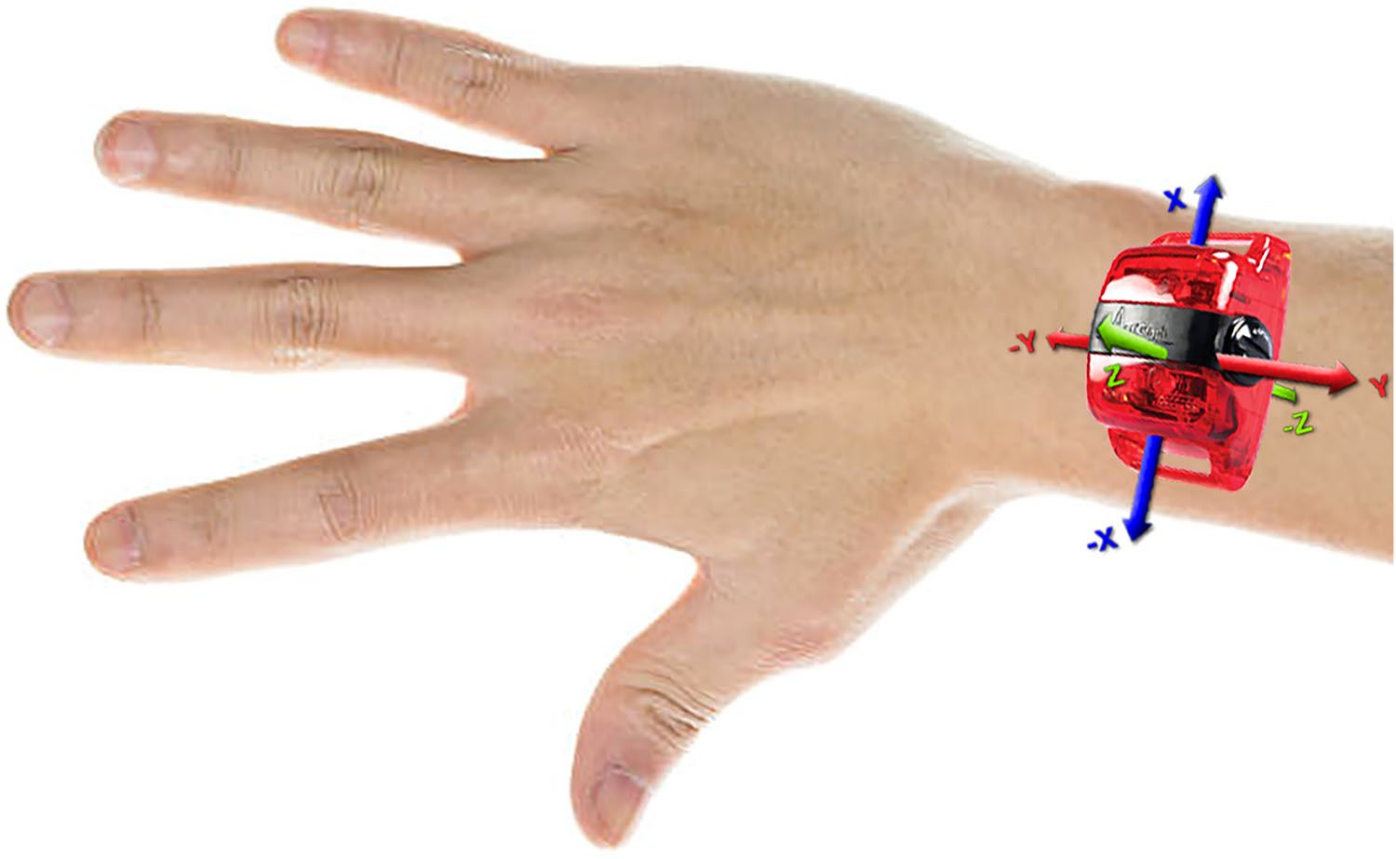
Sensor orientation. Direction of the three acceleration axes (participants were instructed to wear the monitor on the back of the wrist with the + ve y axis pointing up the forearm away from the hand).

The Actigraph sensors were initialised using Actilife software (Fig. 1). Please note that for prosthesis users 1 and 2 (PU1 & PU2), and anatomically intact participants 1 and 2 (AI1 & AI2), the sensors were initialised using Actilife version 5; we then upgraded the software to version 6 for compatibility with the newer sensors for all remaining participants. We are not aware of this having any impact on the way the sensor collects the data. Each sensor was fully charged, then initialised to record data continuously for 7 days at 30 Hz. Participants were asked to wear the sensors on the outside of each wrist. The sensors were labelled as ‘left’ or ‘right’ to inform the participants as to which arm they should be worn on. The sensors were also labelled with an arrow pointing towards the hand to inform participants as to the correct orientation of the sensors (this orientation corresponded to the + ve y axis (Fig. 2) pointing up the forearm away from the hand).

### Data collection

Participants were instructed to wear the sensors throughout the recording period, removing them only when they might get wet. Some participants chose to remove the monitors when sleeping or at other times during the day. Times when each of the sensors was/were reported to have been removed were recorded in the activity diary. When the prosthesis was removed from the arm, the participants were instructed to keep the sensors on both wrists (one remaining on the wrist of the unworn prosthesis, and the other on the participant’s anatomically intact wrist). Therefore, at times when the prosthesis was removed, we would only expect to see activity on the sensor which was worn on the anatomical wrist. Times when the prosthesis was reported to be removed were recorded in the activity diary. It is worth noting that when the prosthesis was removed but carried around, this still showed as activity on the prosthesis worn sensor. At the end of the 7-day recording period, participants were instructed to remove the sensors and return them.

### Data download/pre-processing

Data were downloaded from the sensors using Actilife software (Fig. 1). The raw unprocessed data is stored in a .gt3x file^13^. Please note that for prosthesis users 1 and 2 (PU1 & PU2), and anatomically intact participants 1 and 2 (AI1 & AI2), the .gt3x files were downloaded from the sensors using Actilife version 5; we then upgraded the software to version 6 for compatibility with the newer sensors for all remaining participants and any further processing of the data. We are not aware of this having any impact on the data downloaded into the .gt3x files. In the dataset accompanying this manuscript^11^, we have provided this .gt3x file alongside, for ease of re-use, a .csv file and a .wav file containing the same unprocessed data extracted using the Actilife software.

We used proprietary algorithms within the Actilife software to downsample and filter the raw data, collate it into epochs, and convert it into ‘activity counts’. Further details on these proprietary algorithms have been published by Brond *et al*.^14^. For increased reusability, here we have provided the processed activity count data both with and without Actilife’s low frequency extension filter (developed for compatibility with older accelerometers^12^) and with epochs of 1 second and 60 seconds. For our own previously published analysis^6^ we used the 60 s epoch data with the low frequency extension filter (LFE).

For the 20 prosthesis users the 60 s epoch data (with LFE) from the monitor on the prosthesis were passed through a non-wear algorithm developed to remove the periods when the prosthesis was not worn. We have previously published details of this non-wear algorithm as supplementary material to an earlier paper^6^ and the associated Matlab code is included in this dataset^11^. The dataset also includes the files output from this algorithm with activity counts marked as to whether the prosthesis was calculated to be worn.

### Data analysis

The quality of self-report varied between participants^6^. All complete data from the diaries is included in this dataset, however, please note that the monitor removal data for PU4, PU6, and PU14, the prosthesis removal data for PU5, PU11, and PU14, and the sleep data for PU5 and PU14 are incomplete; additionally, no diary data was provided by PU16. Full details are provided under usage notes.

Please note that the 7-day recording period for participants PU4 and PU5 included a period overnight on 25^th^–26^th^ March 2017 when the clocks in the UK moved forward one hour for British Summertime. This was not accounted for by the internal clock within the activity monitors which was initiated prior to the time change, and therefore the timestamps for the monitor data after this period were 1 hour earlier than any self-reported times in the diaries. To ease interpretation, in this dataset we have manually adjusted the times reported in the diaries to align with the monitor data (thus suggesting that the person reported to start waking up one hour earlier, when in fact this did not occur). More detail on the diary data is provided in the usage notes. If patterns in the timeseries data were to be analysed, another approach could be to offset the activity monitoring data by 1 hour by including a ‘blank’ hour.

Analyses of these data^6^ suggested that anatomically intact participants were symmetrical in their upper limb activity, with only a slight skew of reliance towards the dominant side. Prosthesis users on the other hand showed a preference towards their anatomically intact side during periods where they wore their prosthesis. Patterns of prosthesis wear varied between prosthesis users, with some wearing the prosthesis for extended periods, and others taking it on and off throughout the day. We did not find a correlation between prosthesis wear and prosthesis use, with one of the all-day prosthesis users showing as the least symmetrical participant with respect to their upper limb activity during the prosthesis wear time. We did find that participants who had been prescribed a myoelectric prosthesis for more years tended to use the arms more symmetrically during the times when the prosthesis was worn. It would be interesting to establish whether any other patterns exist, for example do increased prosthesis wear time or time since prescription relate to an increased magnitude of activity from the prosthetic side? This data could also be used to help us to understand if/how movements of a prosthetic arm differ from those of an anatomical arm, and whether there is a relationship between the amount of wear of a prosthesis and the tendency of the movement patterns towards those of an anatomically intact arm.

## Data Records

All of the data described in this manuscript are stored in Figshare^11^.

A metadata file is provided with the following information relating to each participant.

- Gender
- Age
- Sensor type
- Serial number for right sensor
- Serial number for left sensor
- Data records available for each participant (e.g. available/incomplete/missing)
  - Raw .gt3x
  - Raw .csv
  - Raw .wav
  - 60 s epoch activity counts .csv (without low frequency extension filter)
  - 1 s epoch activity counts .csv (without low frequency extension filter)
  - 60 s epoch activity counts .csv (with low frequency extension filter)
  - 1 s epoch activity counts .csv (with low frequency extension filter)
  - Wear data .csv
  - Sleep diary .csv
  - Prosthesis wear diary .csv
  - Sensor wear diary .csv
  For prosthesis users only
- Side of limb absence (and whether this was previously the dominant side)
- Cause of limb absence

For anatomically intact participants only

- Dominance

### Raw accelerometer data

The raw 30 Hz unprocessed data collected on the Actigraph monitors are contained in .gt3x files. Actigraph provide documentation to guide the decoding of the .gt3x file format through github^13^. For ease of re-use, we have also provided this raw accelerometer data in .csv format extracted using the Actilife software and have also converted these .csv files into .wav files using Matlab to allow for faster import. The .wav files have an amplitude range from −1 to 1, therefore, the data contained in the .wav files have been divided by a scaling factor based on the dynamic range of the sensor; to convert these data back to their raw format, the data should be multiplied by 6 (for the GT3X + and wGT3X sensors) or 8 (for the wGT3X-BT sensors).

### Activity counts/epoch data

Data grouped in 1 s and 60 s epochs (with and without the Actilife low frequency extension filter) are provided as .csv files. To create these .csv files, the .gt3x files were first converted into .agd files using the Actilife software, which were then converted into .csv datatables. Each .csv file contains 11 header rows, after which, each row contains the data from one epoch. Columns 1–2 contain the date and time, columns 3–5 contain the activity counts on each of the three axes, column 6 contains the step count according to the Actilife algorithms, column 7 contains the lux readings, columns 8–11 contain inclinometer values, and column 12 contains the vector magnitude values. For our analysis to date we have concentrated on the vector magnitude values, which are a resultant of the activity counts of all three orthogonal axes.

### Non-wear algorithm

The prosthesis wear/non-wear data output from the non-wear algorithm are provided in .csv files. These files do not contain any header rows. Column 1 contains a timestamp (time in minutes since the start of the recording). Columns 2–4 contain the activity counts for axes x, y, and z respectively for the sensor worn on the prosthesis, and column 5 contains the corresponding vector magnitudes. Columns 6–8 contain the activity counts for axes x, y, and z respectively for the sensor worn on the anatomically intact wrist, and column 9 contains the corresponding vector magnitudes. Finally, column 10 contains the categorisation according to the non-wear algorithm, where 1 signifies prosthesis wear and 0 signifies prosthesis non-wear. To analyse only the times when the prosthesis was worn, all rows where column 10 is equal to 0 should be deleted.

### Self-report diary data

The self-reported diary data was transcribed into .csv files. All times are provided in the format DD/MM/YYYY,hh:mm:ss. Please note that where the participants recorded midnight (00:00:00) in the diary, this has been recorded as the 1^st^ timepoint on the subsequent day (for example midnight at the end of 22/10/2017 was recorded as 23/10/2017,00:00:00).

- Diary-MonitorsRemoved.csv contains the periods the sensors were self-reported to be removed from the wrists. Odd numbered rows record the times the sensors were reported to be removed, and even numbered rows record the times the sensors were reported to be put back on. If the sensors were not worn at the time the recording ended, the final row (sensors put back on) contains the time the recording was set to end. All files contain an even number of rows. It has been assumed that when one sensor was removed from the wrist, the other sensor would also be removed, therefore the times in this file refer to the removal of both of the sensors.
- Diary-Sleep.csv contains the periods the participants self-reported to be asleep. Odd numbered rows record the times the participant reported to go to sleep, and even numbered rows record the times the participant reported to wake up. All data collection began in the middle of the day therefore, all files contain an even number of rows.
- Diary-ProsthesisNonWear.csv, which is only provided for the prosthesis users, contains the periods the participants self-reported to remove the myoelectric prosthesis. Odd numbered rows record the times the participant reported to remove the prosthesis, and even numbered rows record the times the participant reported to put the prosthesis back on. If the prosthesis was not worn at the time the recording ended, the final row (prosthesis put back on) contains the time the recording was set to end. Where participants were not wearing the prosthesis at the start of the recording period, the first row (prosthesis removal) contains the time the recording was set to start. All files contain an even number of rows.

Details on the interpretation of any incomplete diaries are provided in the usage notes.

## Technical Validation

The data from four participants (2 anatomically intact and 2 prosthesis users) were used to pilot the study^1^. The raw data from these 4 participants were visually inspected. The data collected from the wrist of the prosthesis would be expected to be of a lower magnitude than the data from the anatomically intact wrist, and bouts of activity would be expected to be shorter and less frequent. Further, the activity on both sensors would be expected to reduce at night. This was confirmed through the visual inspection.

As can be seen in the supplementary material attached to Chadwell *et al*.^6^, during development of the non-wear algorithm, the self-reported prosthesis and sensor wear times were compared against the vector magnitude of the 60 s activity count epochs. Some limitations/inaccuracies to both the algorithm and the self-report data were highlighted. For example:

- The algorithm calculated the prosthesis wear time to be on average 4.4 hours shorter (over 7 days) than the self-reported wear. From visual inspection of the data, it would suggest that for most participants the algorithm detected prosthesis wear more accurately than self-reported. Participants generally appear to over-report their prosthesis wear time.
- The algorithm was unable to differentiate between the prosthesis being carried or sat on a chair in the car, and the prosthesis being worn.
- The algorithm was unable to detect short <20 min periods of prosthesis non-wear.

## Usage Notes

For the prosthesis users, 17 complete sleep diaries were received (2 incomplete, 1 missing), 16 complete prosthesis wear diaries were received (3 incomplete, 1 missing), and 16 complete sensor wear diaries were received (3 incomplete, 1 missing). For the anatomically intact participants, 18 complete sleep diaries were received (1 incomplete, 1 missing), and 20 complete sensor wear diaries were received. Here we report any differences between the .csv files provided with this dataset^11^ and the paper diaries provided by the participants. Note that we have presented these in the format the paper diaries were laid out, rather than in the .csv file layout.

The recording period for two of the prosthesis users (PU4 and PU5) spanned the evening (25^th^–26^th^ March 2017) when the clocks changed by 1 hour due to British Summertime. To align the diary data with the timestamps on the activity data, the reported times were reduced by one hour as demonstrated in Table 1.

**Table 1:** Sleep diary for PU4. Times reported on 26^th^–30^th^ March have been adjusted by 1 hour compared to the self-report to allow direct comparison against the timestamps in the activity monitor data.

	Self-report		Diary file		
	Woke up	Went to sleep	Woke up	Went to sleep	
23-Mar		21:20		21:20	
24-Mar	06:00	20:25	06:00	20:25	
25-Mar	06:00	19:40	06:00	19:40	
26-Mar	06:00	21:30	**05:00**	**20:30**	British Summertime
27-Mar	06:10	21:40	**05:10**	**20:40**	British Summertime
28-Mar	06:00	21:35	**05:00**	**20:35**	British Summertime
29-Mar	06:00	00:05	**05:00**	**23:05**	British Summertime
30-Mar	09:00		**08:00**		British Summertime

**Table 2:** Sensor removal diary for PU4. We were unable to make sense of the self-reported times on 28^th^–30^th^ March when looking at the data, these times have therefore been excluded from the diary file. For this participant, times have also been adjusted for British Summertime as explained in Table 1.

	Self-report		Diary file		
	Removed	Put on	Removed	Put on	
23-Mar	21:00	21:15	21:00	21:15	
24-Mar	20:50	21:16	20:50	21:16	
25-Mar	19:00	19:35	19:00	19:35	
26-Mar	21:20	21:40	**20:20**	**20:40**	British Summertime
27-Mar	21:10	21:35	**20:10**	**20:35**	British Summertime
28-Mar	21:10	06:35	**—**	**—**	British Summertime
29-Mar		06:35		**—**	British Summertime
30-Mar	16:50	09:30	**—**	**—**	British Summertime

**Table 3:** Sleep diary for PU5. Please note that for this participant, times have been adjusted for British Summertime as explained in Table 1. The participant did not report times for going to sleep on 27^th^/28^th^/30^th^, therefore the corresponding waking times have been excluded from the diary file.

	Self-report		Diary file		
	Woke up	Went to sleep	Woke up	Went to sleep	
25-Mar		04:00		04:00	
	11:00	22:15	11:00	22:15	
26-Mar	11:00		**10:00**		British Summertime
27-Mar		01:00		**00:00**	British Summertime
	09:00		**08:00**		British Summertime
28-Mar	10:00		**—**		British Summertime
29-Mar	10:30		**—**		British Summertime
30-Mar		01:15		**00:15**	British Summertime
	10:00		**09:00**		British Summertime
31-Mar	10:50		**—**		British Summertime

**Table 4:** Prosthesis removal diary for PU5. Please note that for this participant, times have been adjusted for British Summertime as explained in Table 1. The data would suggest that the participant was wearing the prosthesis on the first day of recording (24^th^), therefore it is unclear what time it was removed in order to be put back on the morning of the 25^th^, therefore we have excluded this timestamp from the diary file. Additionally, the prosthesis was reported to be removed on the evening of the 28^th^, but as no time was reported for it to be put back on the 29^th^ before being removed again that evening, we have excluded the removal on the 28^th^ from the diary file.

	Self-report		Diary file		
	Put on	Removed	Put on	Removed	
25-Mar	12:30	16:40	**—**	16:40	
	17:45	22:15	17:45	22:15	
26-Mar	13:00	15:38	**12:00**	**14:38**	British Summertime
27-Mar	10:30	22:00	**09:30**	**21:00**	British Summertime
28-Mar	10:30	17:35	**09:30**	**16:35**	British Summertime
	17:38	22:26	**16:38**	**—**	British Summertime
29-Mar		15:20		**14:20**	British Summertime
30-Mar	10:28	11:36	**09:28**	**10:36**	British Summertime
	15:45		**14:45**	**23:31**	British Summertime
31-Mar		00:31	**16:30**		End time

**Table 5:** Sensor removal diary for PU6. The participant did not provide a time for putting the sensor back on the 2^nd^ (instead they put a question mark), therefore, we have excluded the removal time from the diary file.

	Self-report		Diary file	
	Removed	Put on	Removed	Put on
30-Mar	07:09	07:50	07:09	07:50
31-Mar	13:15	13:42	13:15	13:42
02-Apr	10:41	?	**—**	**—**
	20:38	23:01	20:38	23:01
04-Apr	06:47	07:08	06:47	07:08

**Table 6:** Prosthesis removal diary for PU11. The prosthesis was reported to be removed on the evening of the 15^th^, but as no time was reported for it to be put back on the 16^th^ before being removed again that evening, we have excluded the removal on the 15^th^ from the diary file.

	Self-report		Diary file	
	Put on	Removed	Put on	Removed
11-Aug		15:24		15:24
12-Aug	06:27	06:42	06:27	06:42
	09:05	14:25	09:05	14:25
13-Aug	09:20	13:52	09:20	13:52
	15:01	16:54	15:01	16:54
14-Aug	08:30	14:23	08:30	14:23
15-Aug	08:54	15:04	08:54	**—**
16-Aug		12:08		12:08
17-Aug	08:40	18:21	08:40	18:21
18-Aug	08:55		08:55

**Table 7:** Sleep diary for PU14. No times were reported for going to sleep on the 2^nd^, 7^th^, or 8^th^, therefore, we have excluded the corresponding waking times from the diary file.

	Self-report		Diary file	
	Woke up	Went to sleep	Woke up	Went to sleep
03-Aug	08:00	23:00	**—**	23:00
04-Aug	08:00	23:30	08:00	23:30
05-Aug	08:00		08:00	
06-Aug		00:30		00:30
	06:30		06:30	
07-Aug		00:00		00:00
	07:30		07:30	
08-Aug	07:00		**—**	
09-Aug	08:00		**—**

**Table 8:** Prosthesis removal diary for PU14. The data would suggest that the participant was wearing the prosthesis on the first day of recording (2^nd^), therefore it is unclear what time it was removed in order to be put back on in the morning on the 3^rd^, therefore we have excluded this timestamp from the diary file. Additionally, the prosthesis was reported to be put on in the morning on the 8^th^ and 9^th^, but as no time was reported for it to be removed prior to these times we have excluded these timestamps from the diary file.

	Self-report		Diary file	
	Put on	Removed	Put on	Removed
03-Aug	08:00	20:30	**—**	20:30
04-Aug	09:00	21:00	09:00	21:00
05-Aug	08:30	20:00	08:30	20:00
06-Aug	07:30	21:00	07:30	21:00
07-Aug	08:15	16:30	08:15	16:30
	19:50		19:50	
08-Aug	07:30		**—**	
09-Aug	08:40		**—**

**Table 9:** Sensor removal diary for PU14. The sensors were reported to be put on in the mornings on the 8^th^ and 9^th^, but as no corresponding removal times were reported, these timestamps have been excluded from the diary file.

	Self-report		Diary file	
	Removed	Put on	Removed	Put on
02-Aug	22:00		22:00	
03-Aug		08:30		08:30
05-Aug	08:40	09:10	08:40	09:10
07-Aug	16:30	19:50	16:30	19:50
08-Aug		07:30		**—**
09-Aug		08:40		**—**

No sleep data was provided by AI15, therefore the file ‘Diary-Sleep.csv’ for this participant is empty.

Participant AI18 provided no times for going to sleep on the evening of 26^th^ or waking up on the morning of the 27^th^. As they self-reported not to be wearing the sensors during this period, this should not have any impact on the dataset.

Some of the prosthesis users provided incomplete activity diaries. Tables 2–9 compare the difference between the self-reported times and the data included in the diary files with this dataset. Data which have been adjusted or excluded from the diary files are highlighted in bold.

No diary data was provided by PU16, therefore all diary files for this participant are empty.

### Limitations

**Fig. 3:**
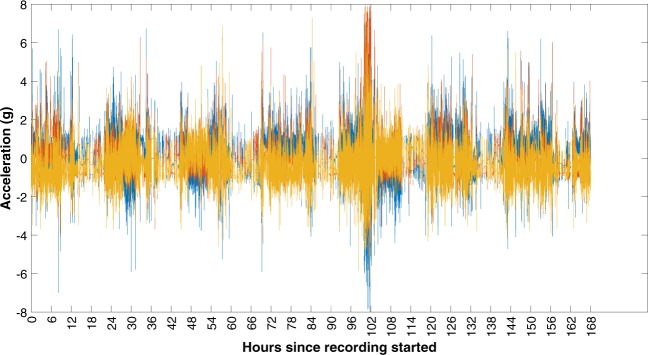
Accelerations recorded with a dynamic range of 8 g. Raw unprocessed tri-axial acceleration data recorded for participant AI19 using a wGT3X-BT sensor with a dynamic range of 8 g. If this data had been collected with a dynamic range of 6 g, clipping would have occurred for some of the signals. It is also worth noting that this was the only participant where possible clipping at 8 g was visible (day 5).

There are some limitations to this dataset which should be considered when reanalysing the data. Due to the availability of sensors, data were collected on different generations of Actigraph sensor. From discussions with Actigraph we do not believe that this has had a major impact on our data. However, it is worth noting that the dynamic range of the accelerometer for the wGT3X-BT was +/−8g whilst for all other generations of the sensor it was +/−6g. This should be considered when evaluating the magnitude of activity collected using the 6 g sensors where clipping may occur (Fig. 3).

It is also worth noting that activity monitoring sensors are liable to offset and clock drift^15,16^. When comparing the data across the two sensors it is therefore possible that the epochs under evaluation do not cover precisely the same time period. This limitation should be considered if the epoch length were to be reduced, or the recording period were to be increased.

As noted previously, for some participants the diary data was incomplete, additionally our analysis suggests that in some cases the self-report under/overestimated the wear time. Further, we highlighted above that for two of the participants the clocks changed to British Summertime during the recording period. This should be taken into consideration if evaluating day-day patterns in activity. It is worth noting that based purely on the data from the activity monitors we are unable to differentiate between the prosthesis/monitors being worn or carried, and the self-report data should therefore also be considered.

Some participants mentioned accidentally wearing the monitors upside down (+ve y axis pointing towards hand), or on the inside of the wrists (for comfort reasons). Our analysis made use of the vector magnitude (resultant) of the acceleration along the three axes, however, if in the re-use of the data the individual axes of acceleration were to be used to determine the orientation of the arm, the data should be sense checked to ensure the orientation of the monitor on the wrist is known. This may be of use if for example a new non-wear algorithm were to be developed where the orientation of the arm was to be used as another factor to establish whether the prosthesis had been removed.

We have only undertaken detailed testing of the prosthesis non-wear algorithm using 60 s epochs, if a different epoch size were to be passed through the algorithm, we would recommend the data is checked against the self-reported non-wear according to the methods published in the supplementary material attached to our associated Scientific Reports paper^6^. It is worth noting that participants only recorded wear times to the nearest minute, so validating the non-wear algorithm for epoch lengths less than 60 s should be approached with considerable caution.

The prosthetic hands used in this dataset contained electric motors which allowed the user to open/close the prosthetic hand. Knowledge of when the prosthetic hand was activated would provide further useful insights into the prosthesis use. It would therefore be interesting in future studies to see whether the activation of the motors in the hand resulted in any detectable patterns within the raw acceleration data recorded at the wrist, or whether additional sensors would be required to record this activity.

It is worth noting that from this dataset we have no information on the use of the affected arm during times when the myoelectric prosthesis was not worn (this was not the goal of the study for which this data was collected). Future studies should also consider the arm activity during these periods when the prosthesis is not worn. Makin *et al*.^17^, have previously collected data during periods when a prosthesis was not worn by placing sensors on the anatomically intact wrist and the proximal aspect of the upper arm on the affected side, however, they did not record prosthesis wear, so it is not possible to compare the activity between wear and non-wear periods. Additionally, by placing the sensors in different locations on each arm, the accelerations are not directly comparable.

Finally, a recent study from Makin’s lab by Maimon Mor^18^ gives us further indication of the potential value of activity monitoring data in this population. In their study, data were collected from accelerometers worn on both the prosthetic and anatomical limbs of people with unilateral upper limb absence whilst performing ‘gesture facilitating tasks’. Activity data were also collected over a 2-day period and the researchers found positive correlations between the amount of involvement of the anatomical limb in the gesture tasks and prosthesis usage; and between prosthesis embodiment and usage. The ability to explore how lab-based data may, or may not correlate with real world use/wear data, provides researchers with a novel approach to the interpretation of both traditionally-used, and novel outcome measures.

## Data Availability

The Matlab code used to detect periods of prosthesis wear/non-wear is included with the dataset on figshare^11^ The inputs to this function are: (1) a 4 column matrix containing the epoch data from the monitor worn on the prosthesis (activity counts on the x, y, and z axes and the vector magnitude), (2) a 4 column matrix containing the epoch data from the monitor worn on the anatomically intact wrist (activity counts on the x, y, and z axes and the vector magnitude), and (3) the epoch size in minutes. Within the function, the prosthesis worn monitor data for each epoch is analysed, and the epoch marked as wear or non-wear. Full details on the algorithm used to determine whether each epoch should be categorised as wear/non-wear have been published previously as supplementary material to Chadwell *et al*.^6^ and in the appendices of Chadwell’s thesis^2^. The algorithm involved comparison of each epoch to the previous epoch. If activity was recorded during a prosthesis non-wear period, or the vector magnitude was equal to 0 during a prosthesis wear period the neighbouring epochs were evaluated to determine whether the prosthesis had been put on/removed. Due to the design of the algorithm, it is unlikely that prosthesis removal or wear periods shorter than 20 minutes would be detected. The output from the function is a 10-column matrix containing the timestamp (minutes since recording began), the data from the two 4 column matrices passed into the function initially, and the categorisation (wear = 1, non-wear = 0). The non-wear algorithm was developed for use with the 60 s epoch vector magnitude data exported from Actilife, however the algorithm has the compatibility for use with other epoch sizes (please note the limitations detailed above). Subsequently to the collection of this dataset, we have also used the non-wear algorithm with data collected using Axivity sensors, where the raw data has been converted into ‘Actigraph activity counts’ using the codes developed by Brond *et al*.^14^. The Matlab code used to produce the spiral plots is not publicly available at this stage, however, by contacting the corresponding author, we would be happy to collaborate to enable you to reproduce your own data. The methods for producing these plots are published in Chadwell *et al*.^8^. Codes have also been developed to allow data from other sensors to be displayed in this format, for example the data from Axivity sensors has been visualised over a 14-day period following similar methods combined with the code developed by Brond *et al*.^14^. These spiral plots also have potential for use with other types of data besides upper limb activity data.

## References

[1] Chadwell, A., Kenney, L., Thies, S., Galpin, A. & Head, J. The reality of myoelectric prostheses: Understanding what makes these devices difficult for some users to control. *Front*. *Neurorobot*. **10**, 10.3389/fnbot.2016.00007 (2016). 10.3389/fnbot.2016.00007 PMC4992705 27597823

[2] Chadwell, A. *The Reality of Myoelectric Prostheses: How do EMG skill*, *unpredictability of prosthesis response*, *and delays impact on user functionality and everyday prosthesis use?*, PhD Thesis, University of Salford, http://usir.salford.ac.uk/id/eprint/47264 (2018).

[3] Gambrell CR (2008). Overuse syndrome and the unilateral upper limb amputee: consequences and prevention. JPO.

[4] Jones LE, Davidson JH (1999). Save that arm: A study of problems in the remaining arm of unilateral upper limb amputees. Prosthet. Orthot. Int..

[5] Østlie K, Franklin RJ, Skjeldal OH, Skrondal A, Magnus P (2011). Musculoskeletal pain and overuse syndromes in adult acquired major upper-limb amputees. Arch. Phys. Med. Rehabil..

[6] Chadwell A (2018). Upper limb activity in myoelectric prosthesis users is biased towards the intact limb and appears unrelated to goal-directed task performance. Sci. Rep..

[7] Actigraph Corp. *ActiGraph White Paper: What is a count?*, (49E Chase Street, Pensacola, FL 32502), https://s3.amazonaws.com/actigraphcorp.com/wp-content/uploads/2017/11/26205758/ActiGraph-White-Paper_What-is-a-Count_.pdf (2008)

[8] Chadwell A (2017). Visualisation of upper limb activity using spirals: A new approach to the assessment of daily prosthesis usage. Prosthet. Orthot. Int..

[9] Paraschiv-Ionescu A, Perruchoud C, Buchser E, Aminian K (2012). Barcoding Human Physical Activity to Assess Chronic Pain Conditions. PLOS ONE.

[10] Lang, C. E., Waddell, K. J., Klaesner, J. W. & Bland, M. D. A Method for quantifying upper limb performance in daily life using accelerometers. *JoVE*, e55673, 10.3791/55673 (2017). 10.3791/55673 PMC5565027 28518079

[11] Chadwell A (2019). Figshare..

[12] Cain KL, Conway TL, Adams MA, Husak LE, Sallis JF (2013). Comparison of older and newer generations of ActiGraph accelerometers with the normal filter and the low frequency extension. Int. J. Behav. Nutr. Phys. Act..

[13] Judge, D. & Maygarden, J. ActiGraph .gt3x file format. *Github*, https://github.com/actigraph/GT3X-File-Format (2014).

[14] Brond JC, Andersen LB, Arvidsson D (2017). Generating ActiGraph counts from raw acceleration recorded by an alternative monitor. Med. Sci. Sports Exerc..

[15] Barreira TV, Zderic TW, Schuna JM, Hamilton MT, Tudor-Locke C (2015). Free-living activity counts-derived breaks in sedentary time: Are they real transitions from sitting to standing?. Gait & Posture.

[16] Clucas, J., White, C., Koo, B., Milham, M. & Klein, A. Assessing actimeters for inclusion in the Healthy Brain Network. *bioRxiv*, 183772, 10.1101/183772 (2017).

[17] Makin TR (2013). Deprivation-related and use-dependent plasticity go hand in hand. eLife.

[18] Maimon Mor, R. O. *et al*. Gesticulation with hand and prosthesis in congenital one-handers and acquired amputees. In *Conference Proceedings: Trent International Prosthetics Symposium* (2019).

